# Rapid production of precisely edited cattle and mice via a single AAV delivered miniature IscB^#^/ωRNA^#^

**DOI:** 10.1038/s41421-026-00871-y

**Published:** 2026-03-17

**Authors:** Lishuang Song, Xuefei Liu, Anqi Di, Di Wu, Chunling Bai, Lige Bu, Dongchao Pei, Jiaru Lei, Zhenting Hao, Guanghua Su, Yuefang Zhao, Zhuying Wei, Shaorong Gao, Guangpeng Li, Lei Yang

**Affiliations:** 1 State Key Laboratory of Reproductive Regulation and Breeding of Grassland Livestock, College of Life Sciences, Inner Mongolia University, Inner Mongolia Autonomous Region, Hohhot, China; 2 Frontier Science Center for Stem Cell Research, School of Life Sciences and Technology, Tongji University, Shanghai, China

**Keywords:** Genomic analysis, Gene expression profiling, Biological techniques

Dear Editor,

Utilizing CRISPR/Cas tools has simplified the creation of genome-edited cells, but the rapid generation of precisely edited animals is challenging, especially in domestic livestock. The widely used Cas endonuclease is *Streptococcus pyogenes* Cas9 (SpCas9, ~1368 amino acids (aa)), which creates double-strand breaks (DSBs) in target DNA sequences^1^. Then, the DSB is repaired through homology-directed repair (HDR) using a template. Although the use of adeno-associated virus (AAV) to deliver CRISPR/Cas has enhanced the generation of knock-out mice, single-AAV delivery of Cas and HDR-template remains a challenge (cargo sizes are severely constrained < 4.8 kb)^2^. Other precise editing strategies, such as base editing and prime editing, require Cas9-fused effectors that further increase cargo sizes, necessitating dual AAV vectors.

Recent studies identified IscB and TnpB as the ancestors of Cas9 and Cas12, respectively, both with a molecular weight (~500 aa) less than half that of the prototypes, making them more amenable to single-AAV delivery^3,4^. Here, we present all-in-one AAV vectors containing the engineered miniature IscB^#^/ωRNA^#^ and a large HDR-template. Using AAV::IscB^#^/ωRNA^#^-HDR particles, we generated precisely knock-in cattle and mice by directly infecting zygotes, eliminating the requirement for micromanipulation steps (Fig. 1a).

**Fig. 1:**
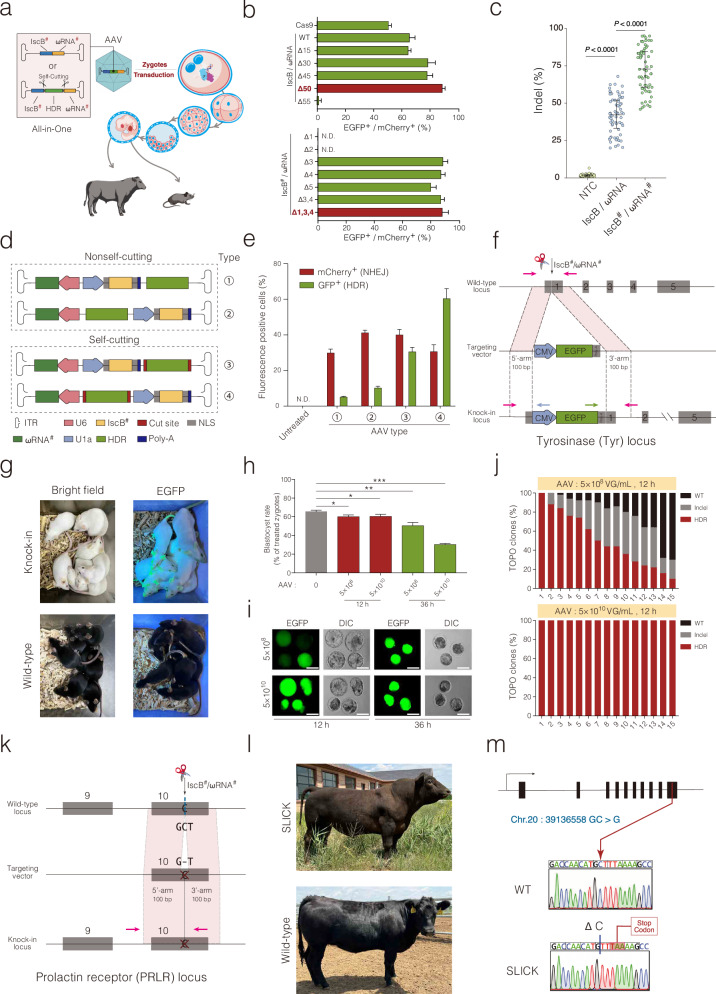
Produce precisely edited mice and cattle by AAV::IscB^#^/ωRNA^#^-HDR. **a** Schematic workflow for precise editing through zygote transduction of AAV::IscB^#^/ωRNA^#^ particles. **b** Comparison of editing activity among IscB, ωRNA, and its variants at the GFxxFP reporters in HEK293T cells. WT wild type; N.D. not detected; mean ± s.d., *n* = 3 independent replicates. **c** Dot-plots showing the overall editing activity of IscB^#^/ωRNA^#^ and prototypical IscB/ωRNA in HEK293T cells. Each dot represents the average editing efficiency of three replicates (*n* = 3) at the endogenous loci. NTC, non-targeting control. The whiskers indicate the upper quartile, mean, and lower quartile values observed across the different loci. *P* values are from Sidak’s multiple comparisons test. **d** Diagram of all-in-one AAV vector containing IscB^#^, ωRNA^#^, and self-cutting HDR-template. ITR, inverted terminal repeats; NLS, nuclear localisation sequence. **e** Comparison of HDR efficiencies of different AAV vectors expressing IscB^#^, ωRNA^#^, and self-cutting HDR-template using the TLR reporters in HEK293T cells. Each dot represents one replicate; mean ± S.D.; *n* = 3 independent replicates; N.D., not detected; NHEJ, non-homologous end joining; HDR, homology-directed repair. **f** Schematic strategy for targeting the Tyr locus. Magenta arrows, Tyr primer; Blue arrow, CMV promoter primer; Green arrow, EGFP primer; pA, short polyA. **g** Representative fluorescence images of pCMV-EGFP knock-in mice generated by AAV::IscB^#^/ωRNA^#^-Tyr/pCMV-EGFP. Wild-type C57BL/6 mouse was used as a control. **h** Bar-graph showing the bovine blastocyst rate, and these embryos were obtained after infecting zygotes with the AAV::IscB^#^/ωRNA^#^/pCMV-EGFP at different concentrations and times. mean ± S.D., *n* = 3 independent replicates; *P < 0.05, **P < 0.01, ***P < 0.001 by two-tailed Student’s *t*-test. **i** Representative fluorescence images of bovine blastocysts obtained by infecting zygotes with AAV::IscB^#^/ωRNA^#^-HDR-EGFP at different doses and durations. *n* = 3 independent replicates; Scale bar, 100 μm. **j** Bar-graphs showing the frequency of HDR-template knock-in using AAV::IscB^#^/ωRNA^#^-HDR-EGFP at different conditions by Sanger sequencing of TOPO-clones. Each bar represents an individual bovine blastocyst, and 50 clones were randomly selected from each embryo for sequencing. **k** Schematic strategy for exon-10 exchange in the PRLR gene. Arrows, primers used for PCR-TOPO sequencing. **l** Representative images of SLICK-cattle generated by AAV::IscB^#^/ωRNA^#^-HDR infection of Angus zygotes. The SLICK-cattle exhibits a characteristic short, slick coat, while the wild-type Angus cattle has a long, thick black coat. **m** Schematic diagram of PRLR-SLICK mutation (chr20:39136558 GC > G) and representative Sanger-sequencing chromatograms.

Given the critical role of the N-terminal in IscB activity^5^, we shortened the C-terminal domain and used an EGXXFP reporter to screen for high-efficiency variants (Supplementary Fig. S1a). Notably, an IscB variant with a 50-aa truncation in the TID-motif (Δ50 aa) showed the highest editing efficiency (~88 vs ~65% of the prototypical type; Fig. 1b). For ωRNA, we truncated individual stem-loops and found that simultaneous deletion of loop-1, loop-3, and loop-4 preserved editing activity (Fig. 1b; Supplementary Figs. S1 and S2). The optimised CRISPR editor, named IscB^#^/ωRNA^#^, was selected for subsequent experiments.

We selected 23 genomic loci for comparison of IscB^#^/ωRNA^#^ and prototypical IscB/ωRNA editing efficiency in HEK293T and HeLa cells (Supplementary Fig. S3a). Amplicon deep sequencing revealed that IscB^#^/ωRNA^#^ exhibited significantly higher overall editing activity in both cell lines: ~73 vs ~42% in HEK293T cells and ~50 vs ~30% in HeLa cells (Fig. 1c; Supplementary Figs. S3b and S4). Engineered IscB^#^/ωRNA^#^ significantly reduced off-target effects compared to prototypical IscB/ωRNA at the top twenty predicted sites (Supplementary Fig. S5). RNA expression levels of IscB^#^/ωRNA^#^ and IscB/ωRNA were similar after transfection, suggesting further optimisation should focus on the structural perspective as engineered with Cas9 (Supplementary Figs. S6 and S7).

Unlike lentiviruses, AAV can penetrate the intact zona pellucida of murine and bovine embryos without prior removal, enabling direct delivery of genetic material into zygotes^6^. To test the feasibility of AAV delivery of IscB^#^ and ωRNA^#^ into intact zygotes for generating knock-out animals, we constructed a single-AAV vector co-expressing IscB^#^ and ωRNA^#^ (AAV::IscB^#^/ωRNA^#^; Fig. 1a). Since tyrosinase (Tyr) is essential for melanin synthesis, we incubated C57BL/6 mice zygotes (black coat) with AAV::IscB^#^/ωRNA^#^ targeting the Tyr gene. Notably, treatment with AAV::IscB^#^/ωRNA^#^-Tyr completely changed the black coat to albino white in all newborn offspring except one (14/15 albino; Supplementary Fig. S8). Amplicon deep sequencing for the Tyr gene revealed that the AAV::IscB^#^/ωRNA^#^ had robust deletion activity (Supplementary Fig. S9). To further confirm the utility of IscB^#^/ωRNA^#^, we compared AAV delivery with conventional microinjection for single- and dual-gene editing and found that AAV delivery achieved comparable single-gene editing efficiencies (84.2 vs 82.1% for Tyr, 74.3 vs 72.5% for Mstn) and feasible dual-gene knockout of Tyr and Mstn (57.1 vs 54.5% mutation rate in both genes), while exhibiting a significantly higher blastocyst formation rate (85.4 vs 65.3%) attributed to the avoidance of microinjection-associated mechanical damage (Supplementary Fig. S10).

To enable precise large-fragment knock-in, we engineered an all-in-one AAV vector containing IscB^#^/ωRNA^#^ and an HDR template of up to ~2.5 kb. Based on previous findings that self-cutting HDR donors (flanked by two CRISPR target sites) enhance HDR efficiency in cells^7^, we used a ‘traffic light reporter (TLR)’ assay to determine the optimal placement of the self-cutting HDR donor within the AAV vector (Fig. 1d, e; Supplementary Fig. S11a)^8^. To validate this approach, we designed a vector targeting Tyr and carrying a pCMV-EGFP HDR template, which was used to infect C57BL/6 zygotes (Fig. 1f). All live-born offspring were albino and expressed green fluorescence (24/24; Fig. 1g), indicating biallelic editing, since a single wild-type Tyr allele would maintain black pigmentation. Consistent with the phenotypic results, genotype analysis also confirmed the correct insertion of the pCMV-EGFP HDR-template at the Tyr locus (Supplementary Fig. S11b, c).

Encouraged by the murine results, we extended the AAV::IscB^#^/ωRNA^#^-HDR system to big livestock cattle. Cattle carrying the SLICK mutation, which involves a single cytosine deletion in exon-10 of the PRLR gene (SLICK-PRLR, chr20:39136558 GC > G), exhibit a short and slick coat that enhances their heat tolerance^9^. To gauge and improve the efficiency of large-fragment knock-in, we first created an AAV-vector containing IscB^#^/ωRNA^#^ targeting PRLR and a pCMV-EGFP HDR-template (Supplementary Fig. S12). Consequently, bovine in vitro fertilisation (IVF) produced zygotes were infected with 5 × 10^8^ or 5 × 10^10^ VG/mL of the above AAV vector for 12 h or 36 h, and then cultured to the blastocyst stage for analysis. Fluorescence and genotype analyses revealed that the combination of a high dose (5 × 10^10^ VG/mL) and short duration (12 h) was the most effective condition, and was selected for subsequent experiments (Fig. 1h–j; Supplementary Fig. S12).

For generating SLICK-cattle via precise exon exchange using AAV::IscB^#^/ωRNA^#^, we constructed an HDR template containing the functional SLICK-PRLR exon-10 (GC > G) flanked by two 100 bp homology arms (Fig. 1k). Infected blastocysts were transplanted into surrogate mothers, resulting in two calves (50%, 2/4), one dead Simmental and one live Angus calf, with an efficiency similar to that of uninfected IVF blastocysts. Indeed, the live Angus calf developed into an adult with a characteristic slick, short coat (Fig. 1l; Supplementary Fig. S13). TOPO-sequencing confirmed successful exon-10 exchange and no off-target effects at the top twenty predicted sites in both calves (Fig. 1m; Supplementary Fig. S14). PCR analysis of ear-tip DNA from the SLICK-cattle and dead calf detected no IscB^#^/ωRNA^#^ signals, indicating that episomal AAV::IscB^#^/ωRNA^#^ genomes were diluted and lost during embryonic development (Supplementary Fig. S15).

Collectively, by systematically truncating the IscB in the TID-motif and ωRNA in the stem-loop, we obtained the miniature IscB^#^/ωRNA^#^ variant with significantly improved editing efficiency. By combining IscB^#^/ωRNA^#^ with a self-cutting HDR-template in an all-in-one AAV vector, we established a micromanipulation-free method for in vitro zygote editing. Encouragingly, we also provide proof-of-principle evidence that the AAV::IscB^#^/ωRNA^#^-HDR system can efficiently produce the so-called SLICK-cattle in one step, which is the first CRISPR animal to be approved as safe by the U.S. Food and Drug Administration (FDA). During the 22 months required for the birth of cattle and development into adulthood, a recent study has shown that deleting the TID-motif at specific positions can convert IscB into an RNA-editing tool^10^, highlighting the versatility of our engineered system for future modifications.

## Supplementary information

Supplementary Information
