# Supplementary material for: Rapid production of precisely edited cattle and mice via a single AAV delivered miniature IscB#/ωRNA#
Source: Cell Discov. 2026 Mar 17;12:18. doi: 10.1038/s41421-026-00871-y (PMC12996356; doi:10.1038/s41421-026-00871-y)
Supplement: Supplementary file 1 — Supplementary Information [file 41421_2026_871_MOESM1_ESM.pdf]

Supplementary information

Rapid production of precisely edited cattle and mice via a single AAV delivered miniature IscB$^{\#}$/ωRNA$^{\#}$

Lishuang Song[1,3], Xuefei Liu[1,3], Anqi Di[1,3], Di Wu[1,3], Chunling Bai[1,3], Lige Bu[1], Dongchao Pei[1], Jiaru Lei[1], Zhenting Hao[1], Guanghua Su[1], Yuefang Zhao[1], Zhuying Wei[1], Shaorong Gao[2,*], Guangpeng Li[1,*], and Lei Yang[1,2,*]

1. State Key Laboratory of Reproductive Regulation and Breeding of Grassland Livestock, College of Life Sciences, Inner Mongolia University, Hohhot 010021, China.
2. Frontier Science Center for Stem Cell Research, School of Life Sciences and Technology, Tongji University, Shanghai 200092, China.
3. These authors contributed equally: Lishuang Song, Xuefei Liu, Anqi Di, Di Wu, Chunling Bai.

* e-mail: gaoshaorong@tongji.edu.cn; gpengli@imu.edu.cn; leiyang@imu.edu.cn

This file includes:

Supplementary Figures 1-17 (Page 2-23)
Supplementary Tables 1-3 (Page 24-32)
Supplementary Methods (Page 33-38)
Supplementary References (Page 39-40)

Supplementary Figures

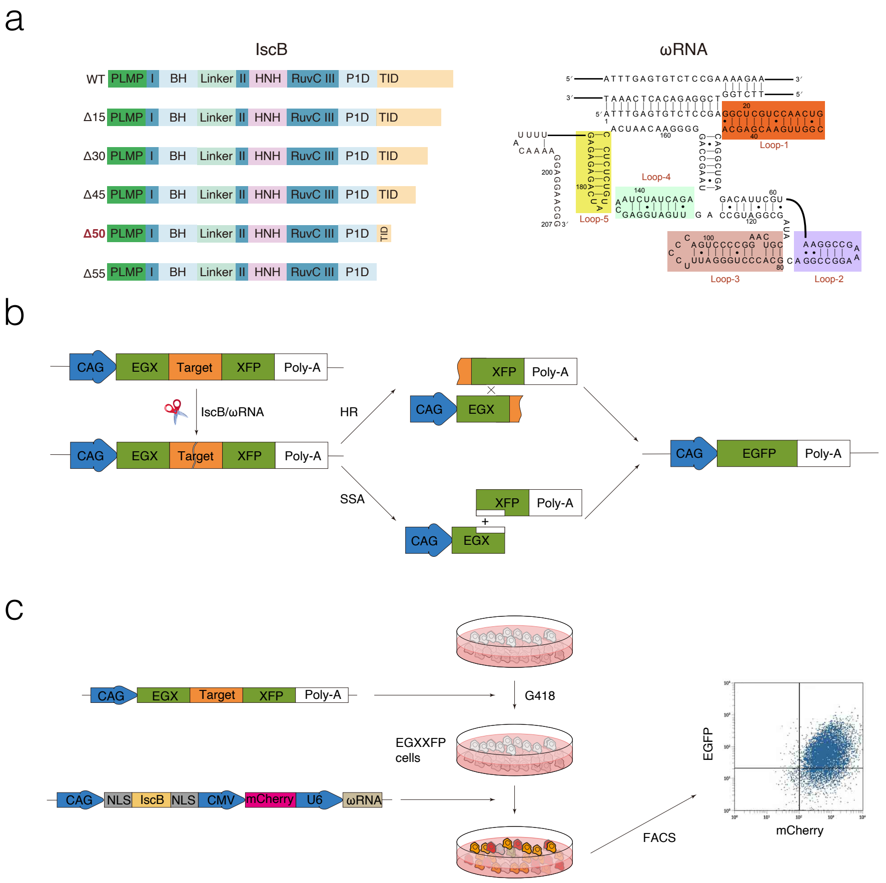

Supplementary Fig. S1 | Workflow of the EGXXFP screen assay for optimizing IscB/ωRNA variants.

a. Schematic diagram of IscB, ωRNA, and its variants with different truncations.

b. The EGXXFP-reporter assay used the pCAG-EGXXFP plasmid, which contains two overlapping 482 bp EGFP fragments under the CAG promoter. A target sequence for endonuclease cleavage is inserted into the EGFP fragments, resulting in a frameshift that abolishes EGFP expression. After endonuclease-mediated target disruption, the frameshifted EGFP can be restored via homologous recombination (HR) or single-strand annealing (SSA), leading to reconstituted EGFP fluorescence. The EGXXFP sequence was adopted from Masahito Ikawa lab (Addgene plasmid # 50716) [1].

c. Schematic overview of the EGXXFP-reporter assay for detecting the cleavage activity of different IscB variants. To generate stable reporter cell lines, HEK293T

cells were transfected with the pCAG-EGXXFP plasmid and selected with geneticin (G418). The EGXXFP-reporter cells were then co-transfected with a plasmid expressing the IscB variant (coupled with mCherry for transfection control) and the corresponding ωRNA targeting the EGXXFP sequence. After 72 hours, the recombinant EGFP fluorescence was measured by fluorescence-activated cell sorting (FACS) analysis.

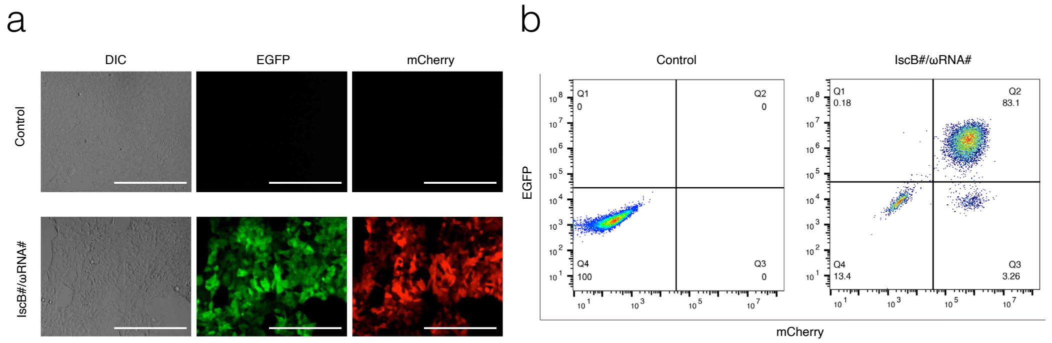

Supplementary Fig. S2 | Expression validation and fluorescence measurement of the EGXXFP-reporter assay.

a. Representative fluorescence images of EGXXFP-reporter cells transfected with IscB$^{\#}$/ωRNA$^{\#}$ for 72 hours. EGXXFP-reporter cells transfected with a blank plasmid (without IscB$^{\#}$/ωRNA$^{\#}$) served as the negative control. Three independent biological replicates were performed with similar results (n = 3); Scale bar, 200 µm.

b. Representative FACS results for the EGXXFP-reporter assay. These images are a representative example of the FACS analysis shown in Fig. 1b. EGXXFP-reporter cells transfected with a blank plasmid (without IscB$^{\#}$/ωRNA$^{\#}$) served as the negative control. Three independent biological replicates were performed with similar results (n = 3).

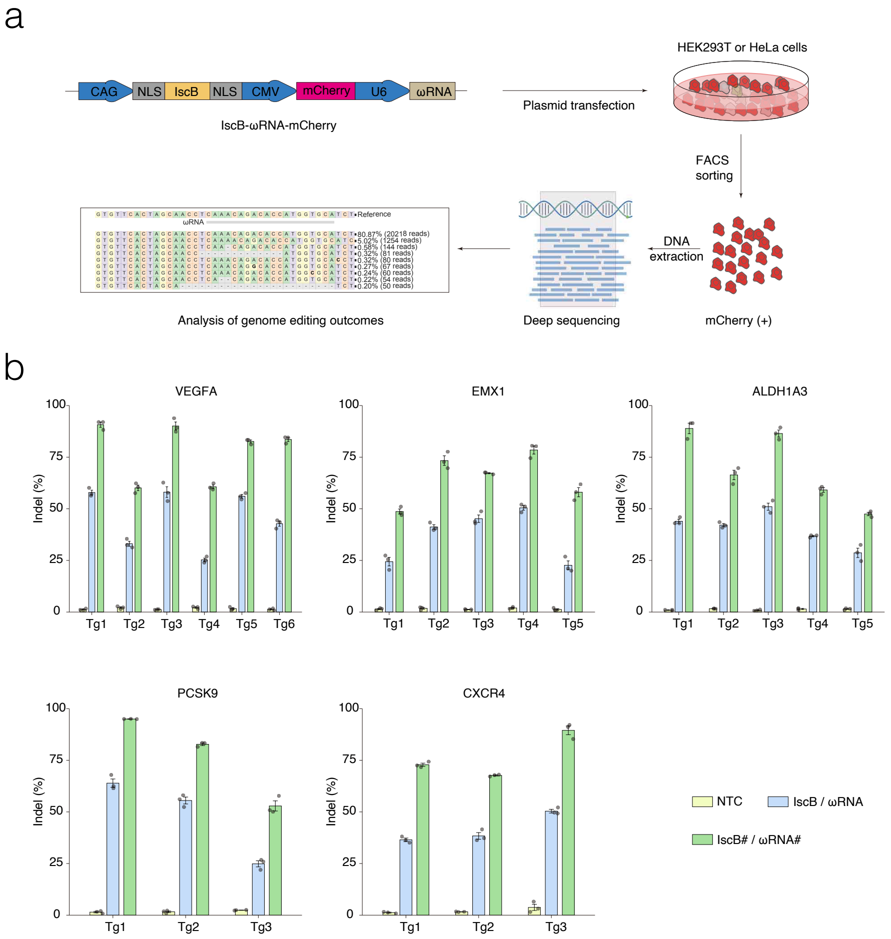

Supplementary Fig. S3 I Schematic and validation of IscB-mediated editing at endogenous genomic loci.

a. Experimental workflow for detecting IscB cleavage activity at endogenous loci. Mammalian cells (HEK293T or HeLa) were transfected with a plasmid encoding IscB, mCherry (transfection marker), and target ωRNA. After 72 hours, the top 25% mCherry-positive cells were sorted by FACS to enrich transfected cells, followed by amplicon deep sequencing to detect the insertions and deletions (indels) at target loci.

b. Comparison of editing efficiencies between IscB#/ωRNA# and prototypical IscB/ωRNA at 22 endogenous genomic loci in HEK293T cells, as determined by amplicon sequencing. NTC, non-targeting control (ωRNA without complementary target sequence); Tg, targeting; Each dot represents one replicate; data are presented as mean ± s.d.; n = 3 independent replicates.

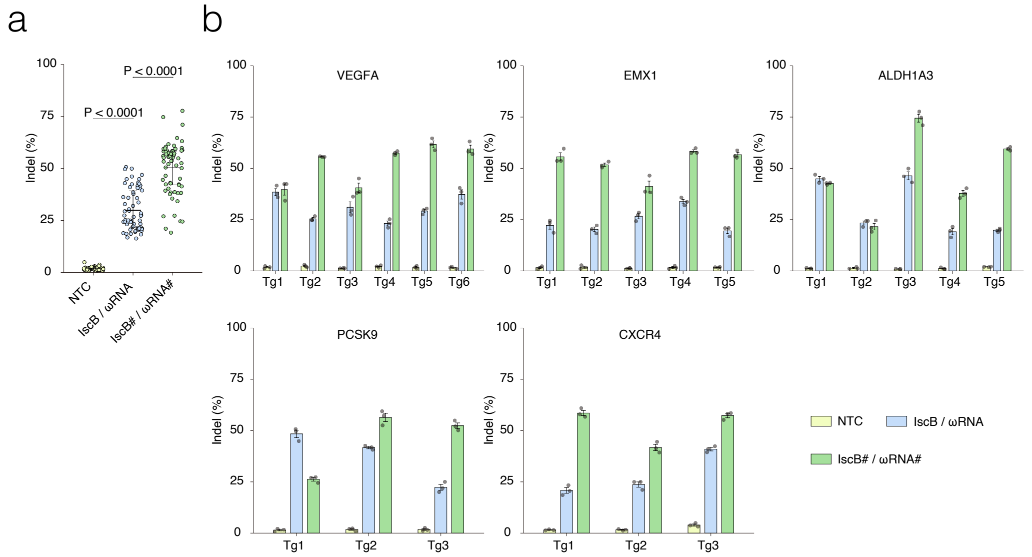

Supplementary Fig. S4 I Validation of IscB$^{\#}$ and IscB editing activity in HeLa cells.

a. Dot-plots showing the overall editing activity of IscB$^{\#}$/ωRNA$^{\#}$ and prototypical IscB/ωRNA in HeLa cells. Each dot represents the average editing efficiency of three replicates (n = 3) at the endogenous loci. NTC, non-targeting control (ωRNA without complementary target sequence); The whiskers indicate the upper quartile, mean, and lower quartile values observed across the different loci.

b. Box-plots showing the overall editing activity of IscB$^{\#}$ and IscB in HeLa cells. Each dot represents one replicate; data are presented as mean ± s.d.; n = 3 independent replicates; NTC, non-targeting control (ωRNA without complementary target sequence); Tg, targeting.

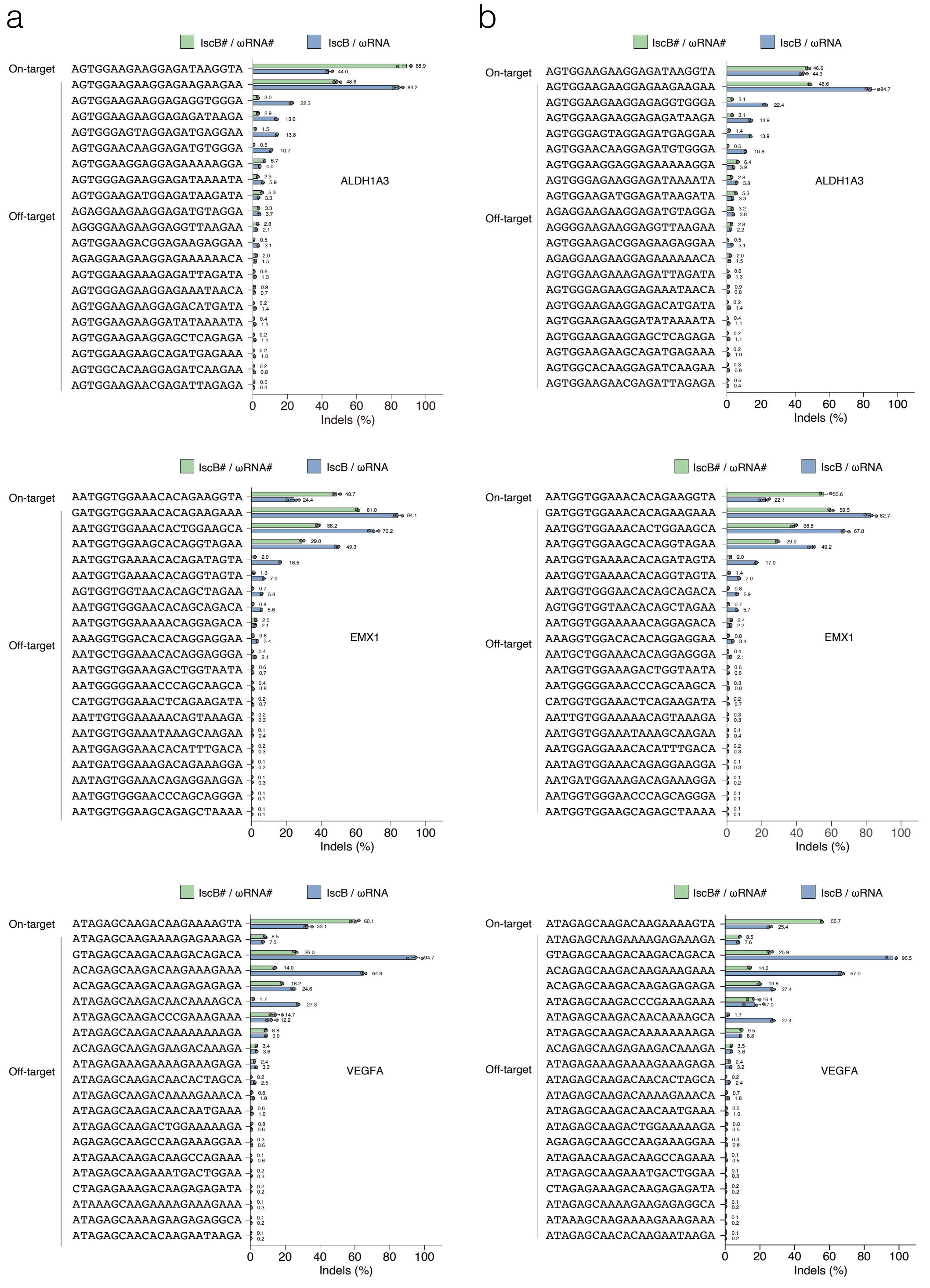

Supplementary Fig. S5 | Off-target effect analysis of IscB# and IscB.

a. Comparison of off-target effects between IscB# and IscB at the top 20 predicted off-target sites in HEK293T cells [2, 3]. Each dot represents one biological replicate; data are presented as mean ± s.d.; n = 3 independent replicates.

b. Comparison of off-target effects between IscB# and IscB at the top 20 predicted off-target sites in HeLa cells [2, 3]. Each dot represents one biological replicate; data are presented as mean ± s.d.; n = 3 independent replicates.

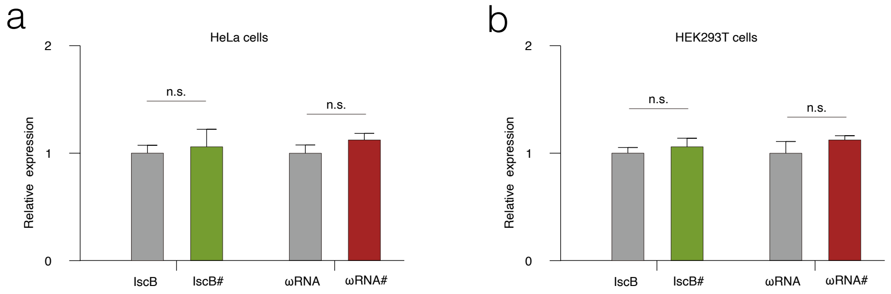

Supplementary Fig. S6 I RNA expression levels of IscB#/ωRNA# and IscB/ωRNA quantified by RT-qPCR.

a. Bar-graphs showing the relative RNA expression levels of IscB#/ωRNA# and IscB/ωRNA quantified by RT-qPCR in HeLa cells. The expression level of IscB andωRNA was set as 1, respectively; data are presented as mean ± s.e.m.; n = 3 independent replicates; n.s., not significant by two-tailed Student's *t*-test.

b. Bar-graphs showing the relative RNA expression levels of IscB#/ωRNA# and IscB/ωRNA quantified by RT-qPCR in HEK293T cells. The expression level of IscB andωRNA was set as 1, respectively; data are presented as mean ± s.e.m.; n = 3 independent replicates; n.s., not significant by two-tailed Student's *t*-test.

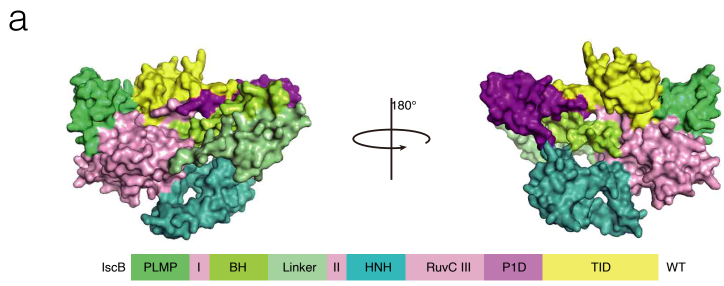

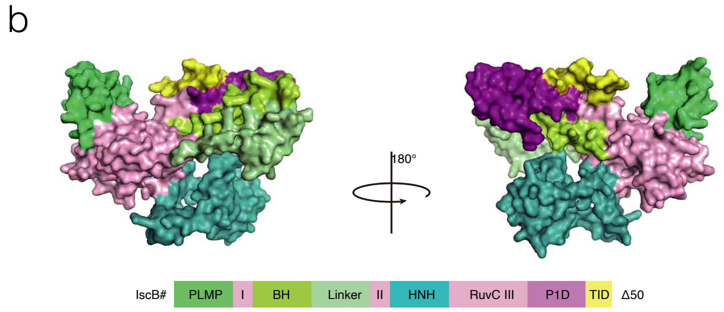

Supplementary Fig. S7 | Structural comparison of IscB# and IscB predicted by AlphaFold2.

a. Predicted structure of IscB by AlphaFold2, displayed in two different orientations (front and side views). The colour scheme is shown at the bottom, with the TID-motif highlighted in yellow.

b. Predicted structure of truncated IscB# (Δ50 aa in TID-motif) by AlphaFold2, displayed in two different orientations (front and side views; corresponding to IscB in a). The colour scheme is shown at the bottom, with the TID-motif highlighted in yellow.

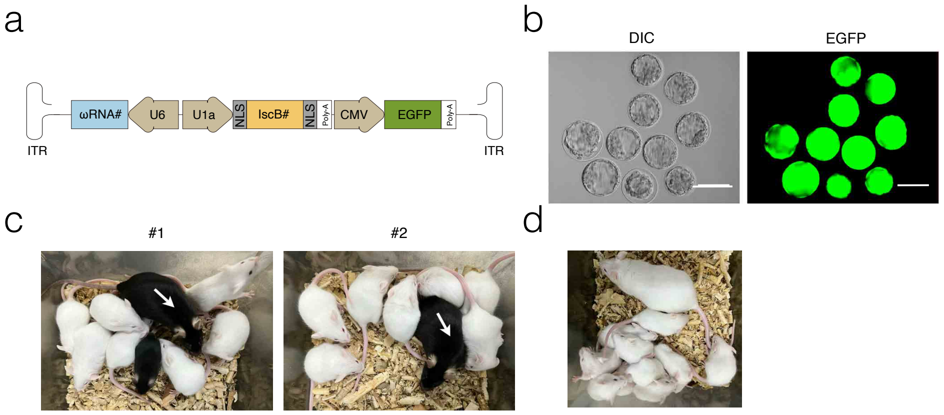

e

Gene editing after transduction of mouse (C57BL/6) zygotes with AAV::IscB#/ωRNA#-Tyr

Treated with	No. of zygotes treated	No. of blastocysts (% of zygotes)	No. of embryos transferred (ET)	No. of ET per recipient	No. of pups at full-term (% of ET)	No. of Tyr-edited pups (albino)	Tyr-editing frequency (% of full-term)
---	25	22 (88.0)	20	10	16 (80.0)	0	0
AAV	27	23 (85.2)	20	10	15 (75.0)	14	93.3

Supplementary Fig. S8 I Efficient gene editing in mouse embryos co-cultured with AAV::IscB#/ωRNA#.

a. Schematic of single-AAV vector expressing IscB#, ωRNA#, and EGFP fluorescence. ITR, inverted terminal repeats; NLS, nuclear localization sequence.

b. Representative fluorescence images of mouse blastocysts obtained after infecting C57BL/6 zygotes with AAV::IscB#/ωRNA#/EGFP-Tyr. Zygotes were incubated for 6 hours in culture medium containing 3×10^9 VG/mL of the AAV vector. Three independent biological replicates were performed with similar results (n = 3). Scale bar, 100 μm.

c. Representative images of two litters derived from zygotes infected with AAV::IscB#/ωRNA#-Tyr. The arrow indicates the surrogate mother. Notably, one wild-type offspring with a black coat was obtained, as zygotes were co-incubated with AAV and directly transferred to the pseudo-pregnant receptor without pre-screening for editing events.

d. Litter derived from a Tyr knock-out male crossed with a wild-type albino CD-1 female. All offspring exhibit a white coat, confirming germline transmission of the Tyr mutation.

e. Summary of Tyr knock-out editing efficiency after transduction of C57BL/6 zygotes with AAV::IscB#/ωRNA#-Tyr vectors.

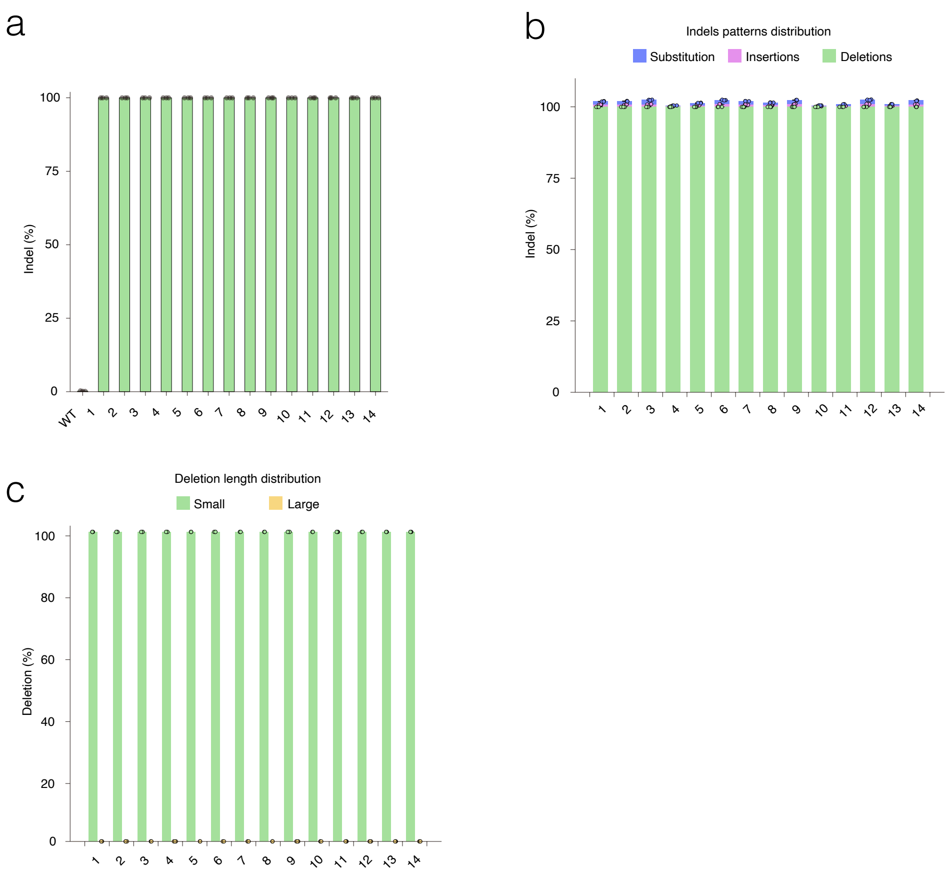

Supplementary Fig. S9 l Further characterisation of indel patterns in Tyr knock-out mice.

a. Bar-graph showing the indel efficiencies of tail DNA from each Tyr knock-out mouse by amplicon sequencing. Wild-type (WT) C57BL/6 mouse was used as a control. Each dot represents one replicate; data are presented as mean ± s.d.; n = 3 independent replicates.

b. Bar-graph showing the indel distribution of tail DNA from each Tyr knock-out mouse by amplicon sequencing. Each dot represents one replicate; data are presented as mean ± s.d.; n = 3 independent replicates.

c. Bar-graph showing the percentages of small (≤ 50 bp) and large (> 50 bp) deletions at the target Tyr locus in each knock-out mouse by amplicon sequencing. Each dot represents one replicate; data are presented as mean ± s.d.; n = 3 independent replicates.

a

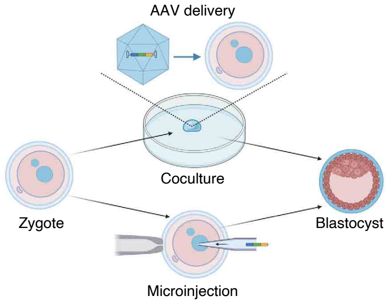

b

Gene editing of mouse (C57BL/6) zygotes using IscB#/ωRNA# through various methods.						
Method	Target gene	No. of zygotes treated	No. of 2-cell embryos (% of treated)	No. of blastocysts (% of treated)	No. of mutant blastocysts with one gene (% of blastocysts)	No. of mutant blastocysts with two genes (% of blastocysts)
---	---	120	112 (93.3)	105 (87.5)	0 (0)	0 (0)
Microinjection	Tyr	121	95 (78.5)	78 (64.5)	64 (82.1)	0 (0)
	Mstn	123	98 (79.7)	80 (65.0)	58 (72.5)	0 (0)
	Tyr + Mstn	118	95 (80.5)	77 (65.3)	Tyr: 62 (80.5) or Mstn: 54 (70.1)	Tyr + Mstn: 42 (54.5)
AAV	Tyr	119	110 (92.4)	101 (84.9)	85 (84.2)	0 (0)
	Mstn	124	116 (93.5)	105 (84.7)	78 (74.3)	0 (0)
	Tyr + Mstn	123	114 (92.7)	105 (85.4)	Tyr: 87 (82.9) or Mstn: 76 (72.4)	Tyr + Mstn: 60 (57.1)

Supplementary Fig. S10 | Feasibility of AAV::IscB#/ωRNA# mediated multiplex editing and comparison with pronuclear microinjection.

a. Schematic diagram of gene editing mediated by IscB#/ωRNA# via two delivery methods: AAV transduction (zygote incubation with AAV::IscB#/ωRNA# particles, no micromanipulation) and pronuclear microinjection (direct injection of IscB#/ωRNA# mRNA into the male pronucleus).

b. Summary of single-site or multiple-site gene editing mediated by IscB#/ωRNA# through AAV delivery or microinjection in mouse embryos. Notably, AAV delivery of IscB#/ωRNA# achieved efficient dual-gene knockout of Tyr and Mstn in mouse zygotes (57.1% of blastocysts with mutations in both genes), showing comparable editing efficiency to microinjection (54.5%); importantly, AAV delivery yielded a much higher blastocyst formation rate (85.4% vs. 65.3% for microinjection), which is attributed to the avoidance of mechanical damage associated with direct microinjection.

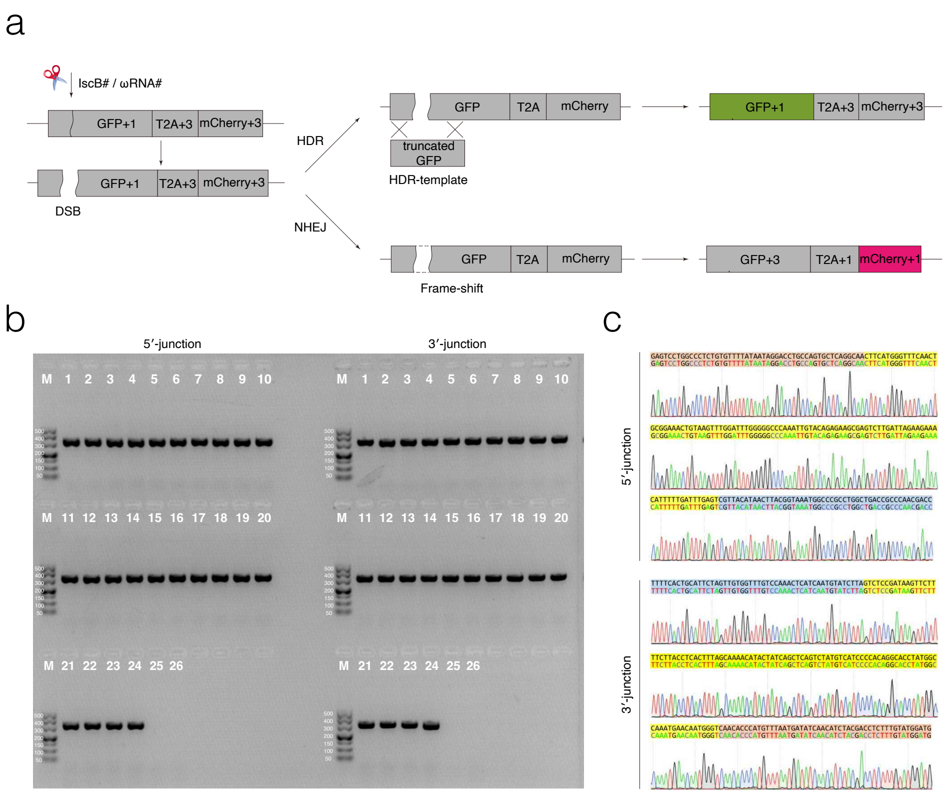

Supplementary Fig. S11 | Characterisation of precise knock-in editing by infecting mouse zygotes with AAV::IscB$^{\#}$/ωRNA$^{\#}$-HDR.

a. Schematic overview of the TLR (traffic light reporter) assay for detecting the HDR activity of different AAV::IscB$^{\#}$/ωRNA$^{\#}$ self-cutting HDR variants. The TLR plasmid contains a disrupted GFP gene (frameshifted) and downstream mCherry gene. If double-stranded breaks (DSBs) are resolved via HDR, the full GFP sequence is reconstituted (green fluorescent cells); if DSBs undergo non-homologous end joining (NHEJ), GFP remains frameshifted and mCherry is expressed (red fluorescent cells). The TLR reporter system was obtained from a previous report[4].

b. Representative of PCR-genotyping of pCMV-EGFP knock-in mice. The left panel shows PCR-amplification of the 5′-junction of the targeted Tyr locus using a forward primer that binds to genomic DNA upstream of the homology region and a reverse primer that binds to the donor HDR-template. The right panel shows PCR-amplification of the 3′-junction of the Tyr-edited allele using a forward primer that binds to the donor HDR-template and a reverse primer that binds to genomic DNA downstream of the homology region. Lanes 1-24: knock-in mice; Lane 25:

wild-type mouse; Lane 26: H_2O; M, DNA marker. The primer binding sites are depicted in Fig. 1f.

c. Representative Sanger sequencing chromatograms of 5′- and 3′-junctional regions in pCMV-EGFP knock-in mice. Pink box, 5′ or 3′ genomic region; Yellow box, 5′ or 3′ homology arm region; Blue box, CMV promoter or EGFP poly-A region. Sequences confirm precise HDR-mediated insertion of the pCMV-EGFP template into the Tyr locus. Primer binding sites are depicted in Fig. 1f.

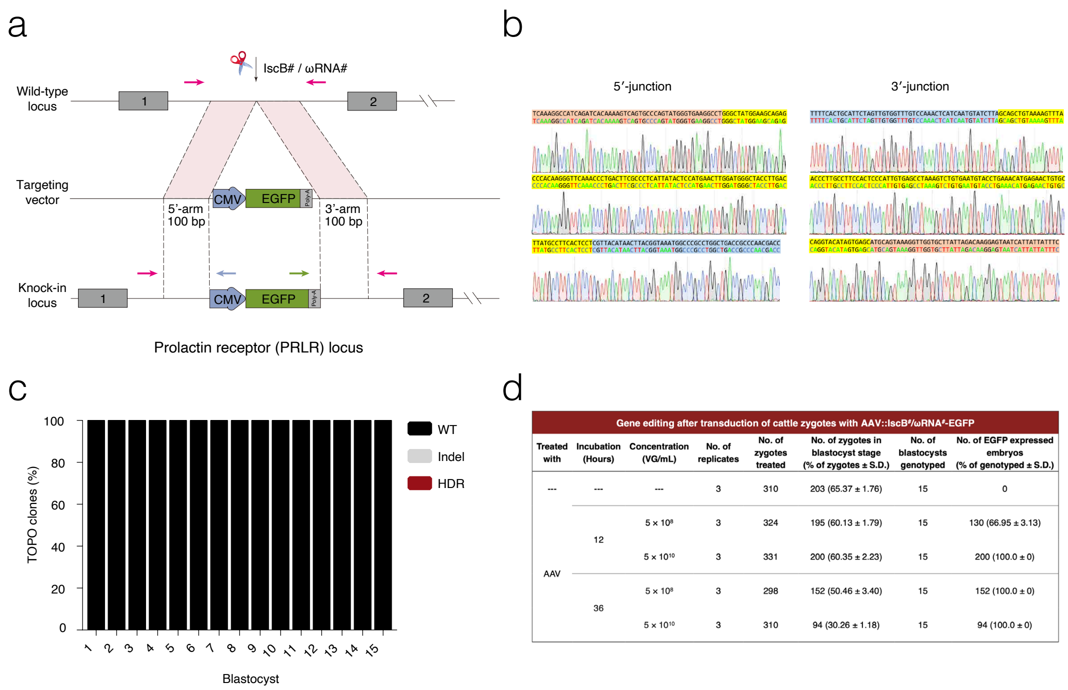

Gene editing after transduction of cattle zygotes with AAV::IscB#/ωRNA#-EGFP							
Treated with	Incubation (Hours)	Concentration (VG/mL)	No. of replicates	No. of zygotes treated	No. of zygotes in blastocyst stage (% of zygotes ± S.D.)	No. of blastocysts genotyped	No. of EGFP expressed embryos (% of genotyped ± S.D.)
---	---	---	3	310	203 (65.37 ± 1.76)	15	0
AAV	12	5×10^8	3	324	195 (60.13 ± 1.79)	15	130 (66.95 ± 3.13)
		5×10^{10}	3	331	200 (60.35 ± 2.23)	15	200 (100.0 ± 0)
	36	5×10^8	3	298	152 (50.46 ± 3.40)	15	152 (100.0 ± 0)
		5×10^{10}	3	310	94 (30.26 ± 1.18)	15	94 (100.0 ± 0)

Supplementary Fig. S12 I Characterisation of precise knock-in editing by infecting bovine zygotes with AAV::IscB#/ωRNA#-HDR.

a. Schematic strategy for targeted knock-in at the bovine PRLR locus. Magenta arrows, PRLR primer; Blue arrow, CMV promoter primer; Green arrow, EGFP primer; pA, short polyA.

b. Representative Sanger sequencing chromatograms of 5′ and 3′ junctional regions in pCMV-EGFP knock-in bovine blastocysts. Pink box, 5′ or 3′ genomic region; Yellow box, 5′ or 3′ homology arm; Blue box, CMV promoter or EGFP poly-A region. Sequences confirm precise HDR-mediated insertion of the EGFP gene into the PRLR locus. Primer binding sites are depicted in Supplementary Fig. S12a.

c. Sanger sequencing was performed on TOPO-clones to analyse mutations at the PRLR locus in AAV-untreated embryos. Each bar represents an individual bovine blastocyst, and 50 clones were randomly selected from each embryo for sequencing. The percentage is calculated as (number of HDR-positive clones / total Sanger-sequenced clones) × 100. WT, wild type.

d. Summary of EGFP knock-in editing efficiency after transduction of bovine zygotes with AAV::IscB#/ωRNA#-EGFP vectors.

a

Pre-implantation development of bovine zygotes						
Treated with	No. of zygotes treated	% 2-cell per treated ± S.D.	% 4-cell per treated ± S.D.	% 8-cell per treated ± S.D.	% morula per treated ± S.D.	% blastocyst per treated ± S.D.
---	120	85.92 ± 2.88	81.59 ± 2.84	78.52 ± 2.50	68.41 ± 1.61	65.71 ± 1.46
AAV	221	84.84 ± 1.31	80.57 ± 2.98	76.70 ± 2.18	62.45 ± 2.88 *	59.63 ± 2.09 *

*$P < 0.05$ as compared with the untreated group, by two-tailed Student's *t*-test.

b

Post-implantation development of bovine zygotes								
Treated with	No. of zygotes treated	No. of zygotes in blastocyst stage (% of treated)	No. of embryos transferred (ET)	No. of ET per recipient	No. of calves (% of ET)	No. of PRLR-edited calves (Slick)	PRLR-editing frequency (% of ET)	No. of calves surviving to adulthood (% of ET)
---	12	8 (66.7)	2	1	2 (100.0)	0	0	2
AAV	10	6 (60.0)	2	1	2 (100.0)	2	2 (100.0)	1

c

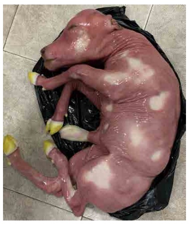

d

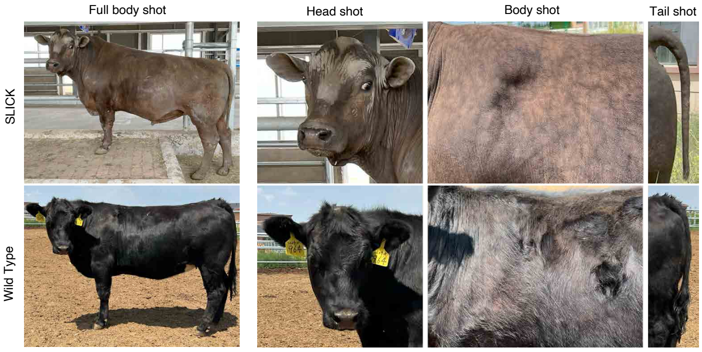

Supplementary Fig. S13 I Further characterisation of the SLICK-cattle phenotype.

a. Summary of pre-implantation development of SLICK-embryos derived by AAV::IscB#/ωRNA#-HDR infection of IVF zygotes. Untreated IVF zygotes were used as the control.

b. Summary of post-implantation development of SLICK-embryos derived by AAV::IscB#/ωRNA#-HDR infection of IVF zygotes. Untreated IVF zygotes were used as the control.

c. Image of a dead Simmental fetus derived by AAV::IscB#/ωRNA#-HDR infection of IVF zygotes. Post-mortem analysis revealed no gross abnormalities, and genotyping confirmed successful SLICK-PRLR exon-10 exchange.

d. Representative images showing coat differences between SLICK-cattle and wild-type Angus cattle. SLICK-cattle exhibit a short, sleek hair coat (adapted to hot environments), while wild-type cattle have a long, thick black hair coat. The difference in coloration between SLICK cattle and wild-type cattle is due to SLICK

cattle having a short, sleek hair coat, while wild-type cattle have long, black coats. Consequently, SLICK cattle show different skin color, whereas wild-type cattle maintain the black hair coat phenotype.

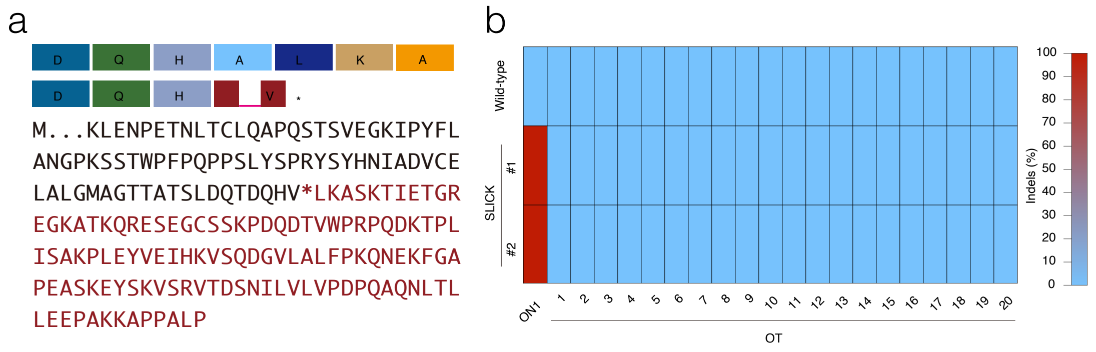

Supplementary Fig. S14 I Genotypic characterisation of SLICK-cattle.

a. Schematic of the 200 C-terminal amino acids of PRLR and SLICK-PRLR isoform. The SLICK-PRLR mutation is a single-base C deletion in the exon of PRLR (chr20:39136558 GC > G), which introduces a premature stop codon (p.Leu462*) and causes the loss of 120 C-terminal amino acids from the PRLR proteins. Red letters, truncated amino acids due to the p.Leu462* mutation.

b. Heat-map showing the off-target effects of IscB#/ωRNA# targeting the PRLR locus at the top twenty predicted sites by TOPO-sequencing. Fifty clones were randomly selected from each cattle for Sanger sequencing. ON, on-target site; OT, predicted off-target site. Wild-type cattle were used as the control.

a

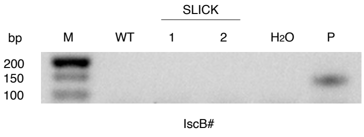

b

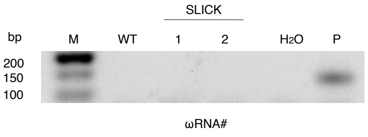

Supplementary Fig. S15 l Identification of AAV::IscB#/ωRNA# in the SLICK-cattle genome.

a. Genomic PCR showing the absence of IscB# in the SLICK-cattle produced by AAV infection of IVF zygotes. The positive and negative controls were amplified from the plasmid and water, respectively. Lane 1: DNA marker; Lane 2: wild-type cattle; Lanes 3-4: SLICK-cattle; Lane 5: H_2O; Lane 6: plasmid. Three independent replicates with the same results (n = 3).

b. Genomic PCR showing the absence of ωRNA# in the SLICK-cattle produced by AAV infection of IVF zygotes. The positive and negative controls were amplified from the plasmid and water, respectively. Lane 1: DNA marker; Lane 2: wild-type cattle; Lanes 3-4: SLICK-cattle; Lane 5: H_2O; Lane 6: plasmid. Three independent replicates with the same results (n = 3).

a

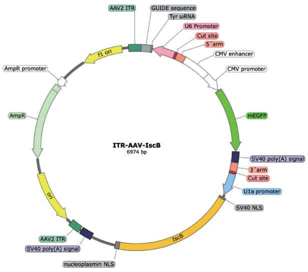

b

ITR-GUIDE sequence-ωRNA#-U6 promoter-Cut site-5´arm-CMV enhancer-CMV promoter-mEGFP-SV40 poly(A) signal-3´arm-Cut site-U1a promoter-SV40 NLS-IscB#-nucleoplasmin NLS-SV40 poly(A) signal-ITR

```
CCTGCAGGCAGCTGCGCGCTCGCTCGCTCACTGAGGCCGCCCGGGCAAAGCCCGGGCGTCGG
GCGACCTTTGGTCGCCCGGCCTCAGTGAGCGAGCGAGCGCGCAGAGAGGGAGTGGCCAACTC
CATCACTAGGGGTTCCTCCGTTCCTCCTTTTGTAAAACTCTCTCTGATACAGAGAGGTGATT
GTTCCCCCTGGCTTACTGGCATCCGCTATGTCCGGCCTTTCGGCCTTACGAATGTCTCAGCC
TGTCGGAGACACTCAAATCCGGTGTTTCGTCCTTTCCACAAGATATATAAAGCCAAGAAATC
GAAATACTTTCAAGTTACGGTAAGCATATGATAGTCCATTTTAAAACATAATTTTAAAACTG
CAAACTACCCAAGAAATTATTACTTTCTACGTCACGTATTTTGTACTAATATCTTTGTGTTT
ACAGTCAAATTAATTCCAATTATCTCTCTAACAGCCTTGTATCGTATATGCAAATATGAAGG
AATCATGGGAAATAGGCCCTCACGCGTATTTGAGTGTCTCCGAAAAGAACTTCATGGGTTTC
AACTGCGGAAACTGTAAGTTTGGATTTGGGGGCCCAAATTGTACAGAGAAGCGAGTCTTGAT
TAGAAGAAACATTTTTGATTTGAGTCGTTACATAACTTACGGTAAATGGCCCGCCTGGCTGA
CCGCCCAACGACCCCCGCCCATTGACGTCAATAATGACGTATGTTCCCATAGTAACGCCAAT
AGGGACTTTCCATTGACGTCAATGGGTGGAGTATTTACGGTAAACTGCCCACTTGGCAGTAC
ATCAAGTGTATCATATGCCAAGTACGCCCCCTATTGACGTCAATGACGGTAAATGGCCCGCC
TGGCATTATGCCCAGTACATGACCTTATGGGACTTTCCTACTTGGCAGTACATCTACGTATT
AGTCATCGCTATTACCATGGTGATGCGGTTTTGGCAGTACATCAATGGGCGTGGATAGCGGT
TTGACTCACGGGGATTTCCAAGTCTCCACCCCATTGACGTCAATGGGAGTTTGTTTTGGCAC
CAAAATCAACGGGACTTTCCAAAATGTCGTAACAACTCCGCCCCATTGACGCAAATGGGCGG
TAGGCGTGTACGGTGGGAGGTCTATATAAGCAGAGCTGCCACCATGGTGAGCAAGGGCGAGG
AGCTGTTCACCGGGGTGGTGCCCATCCTGGTCGAGCTGGACGGCGACGTAAACGGCCACAAG
TTCAGCGTGTCCGGCGAGGGCGAGGGCGATGCCACCTACGGCAAGCTGACCCTGAAGTTCAT
CTGCACCACCGGCAAGCTGCCCGTGCCCTGGCCCACCCTCGTGACCACCCTGACCTACGGCG
TGCAGTGCTTCAGCCGCTACCCCGACCACATGAAGCAGCACGACTTCTTCAAGTCCGCCATG
CCCGAAGGCTACGTCCAGGAGCGCACCATCTTCTTCAAGGACGACGGCAACTACAAGACCCG
CGCCGAGGTGAAGTTCGAGGGCGACACCCTGGTGAACCGCATCGAGCTGAAGGGCATCGACT
TCAAGGAGGACGGCAACATCCTGGGGCACAAGCTGGAGTACAACTACAACAGCCACAACGTC
TATATCATGGCCGACAAGCAGAAGAACGGCATCAAGGTGAACTTCAAGATCCGCCACAACAT
CGAGGACGGCAGCGTGCAGCTCGCCGACCACTACCAGCAGAACACCCCCATCGGCGACGGCC
CCGTGCTGCTGCCCGACAACCACTACCTGAGCACCCAGTCCAAGCTGAGCAAAGACCCCAAC
GAGAAGCGCGATCACATGGTCCTGCTGGAGTTCGTGACCGCCGCCGGGATCACTCTCGGCAT
GGACGAGCTGTACAAGTAAAACTTGTTTATTGCAGCTTATAATGGTTACAAATAAAGCAATA
GCATCACAAATTTCACAAATAAAGCATTTTTTTCACTGCATTCTAGTTGTGGTTTGTCCAAA
CTCATCAATGTATCTTAGTCTCCGATAAGTTCTTTTCTTACCTCACTTTAGCAAAACATACT
ATCAGCTCAGTCTATGTCATCCCCACAGGCACCTATGGCCAAATGAACAATGGGTATTTGAG
TGTCTCCGAAAAGAACTCGAGATGGAGGCGGTACTATGTAGATGAGAATTCAGGAGCAAACT
GGGAAAAGCAACTGCTTCCAAATATTTGTGATTTTTACAGTGTAGTTTTGGAAAAACTCTTA
GCCTACCAATTCTTCTAAGTGTTTTAAAATGTGGGAGCCAGTACACATGAAGTTATAGAGTG
TTTTAATGAGGCTTAAATATTTACCGTAACTATGAAATGCTACGCATATCATGCTGTTCAGG
CTCCGTGGCCACGCAACTCATACTGACGGGAAATGACCGGTGCGGCCGCGATCTCCAAAAAA
GAAACGGAAGGTGATGATGGCCGTGGTGTACGTGATCAGCAAGTCTGGCAAACCCCTGATGC
CCACCACCCGGTGCGGCCACGTGAGAATCCTGCTGAAGGAGGGCAAGGCCAGAGTGGTGGAG
CGGAAACCGTTCACCATCCAGCTGACCTACGAGAGCGCGGAGGAAACACAGCCTCTGGTCCT
GGGCATTGATCCTGGACGCACCAATATCGGCATGAGCGTGGTGACAGAATCCGGAGAGAGCG
TGTTCAACGCCCAGATCAGAACCAGAAACAAGGACGTGCCTAAGCTGATGAAAGACAGAAAG
CAGTACCGGATGGCTCACAGACGGCTGAAGCGGCGCTGCAAGCGAAGAAGAAGAGCCAAGGC
CGCCGGCACCGCATTCGAGGAAGGAGAAAAGCAGAGACTGCTGCCTGGCTGTTTCAAGCCTA
TCACATGTAAGAGCATCAGAAATAAGGAAGCTAGATTCAATAACAGGAAGCGGCCTGTGGGA
TGGCTGACCCCAACCGCCAACCACCTGCTGGTGACCCACCTGAACGTGGTCAAGAAGGTGCA
AAAGATCCTGCCTGTGGCCAAGGTGGTGCTGGAACTGAATCGGTTCAGCTTCATGGCCATGA
ACAACCCCAAGGTGCAGCGGTGGCAGTACCAGAGAGGCCCTCTGTACGGTAAAGGCAGCGTG
GAAGAGGCCGTGAGCATGCAGCAGGACGGCCACTGCCTGTTTTGCAAGCACGGCATTGACCA
CTATCACCATGTGGTTCCTAGGAGAAAGAACGGCTCTGAAACCCTGGAGAACCGGGTGGGCC
TGTGCGAGGAACACCACCGGCTCGTCCACACAGATAAGGAGTGGGAGGCCAATCTGGCTTCT
AAGAAGAGCGGAATGAACAAGAAATACCACGCCCTGAGCGTGCTGAACCAGATCATCCCATA
CCTGGCCGACCAGCTTGCCGATATGTTCCCCGGCAACTTCTGTGTGACCAGCGGACAGGATA
CATACCTGTTTCGGGAAGAACATGGCATCCCCAAGGACCACTACCTGGATGCCTACTGCATC
GCCTGCAGCGCCCTGACCGACGCCAAGAAGGTGAGCAGCCCTAAAGGCAGACCTTACATGGT
GAGACAGTTCAGACGCCACGATAGACAGGCCTGTCACAAGGCCAACCTGAACAGAAGATACT
ACATGGGCGGCAAACTGGTTGCTACAAACAGACATAAGGCCATGGACCAGAAAACCGACAGC
CTGGAGGAGTACAGAGCCGCCCACAGCGCCGCTGACGTATCCAAGCTGACAGTGAAGCACCC
TTCTGCTCAATATAAGGACATGTCCCGGATCATGCCTGGCAGCATCCTCAAAAGGCCGGCGG
CCACGAAAAAGGCCGGCCAGGCAAAAAAGAAAAAGGGATCTGAATTCGGTAGTGGGAAGCTT
GATAATCAACCTCTGGATTACAAAATTTGTGAAAGATTGACTGGTATTCTTAACTATGTTGC
TCCTTTTACGCTATGTGGATACGCTGCTTTAATGCCTTTGTATCATGCTATTGCTTCCCGTA
TGGCTTTCATTTTCTCCTCCTTGTATAAATCCTGGTTAGTTCTTGCCACGGCGGAACTCATC
GCCGCCTGCCTTGCCCGCTGCTGGACAGGGGCTCGGCTGTTGGGCACTGACAATTCCGTGGT
GTTAACTTGTTTATTGCAGCTTATAATGGTTACAAATAAAGCAATAGCATCACAAATTTCAC
AAATAAAGCATTTTTTTCACTGCATTCTAGTTGTGGTTTGTCCAAACTCATCAATGTATCTT
ATCATGTCTGGATCGGCGCGCCAGCGGCCGCAGGAACCCCTAGTGATGGAGTTGGCCACTCC
CTCTCTGCGCGCTCGCTCGCTCACTGAGGCCGGGCGACCAAAGGTCGCCCGACGCCCGGGCG
GCCTCAGTGAGCGAGCGAGCGCGCAGCTGCCTGCAGG
```

Supplementary Fig. S16 | Sequence information of all-in-one AAV plasmid.
Circular map (a) and sequence information (b) of AAV::IscB#/ωRNA#-HDR plasmid. ITR, inverted terminal repeats; U6 promoter, the Pol III promoter to express small RNA; Cut site, a donor HDR DNA with flanking ωRNA target sites; 5’ arm, the 5’ homology arm in the HDR donor; CMV promoter, cytomegalovirus (CMV) promoter; CMV enhancer, CMV immediate-early enhancer; mEGFP, monomeric enhanced green fluorescent protein; SV40 poly(A), Simian virus 40 PolyA; 3’ arm, the 3’ homology arm in the HDR donor; U1a promoter, the small nuclear RNA promoter; NLS, nuclear localization signal.

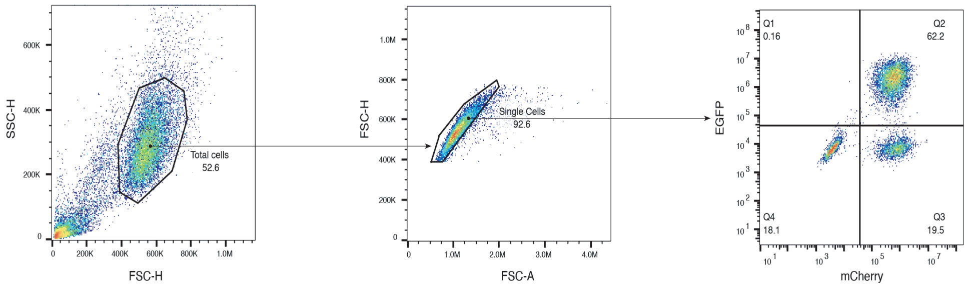

Supplementary Fig. S17 l FACS gating examples for the EGXXFP-reporter assay. FACS gating examples for the EGXXFP-reporter assay, according to the published protocols (details in Supplementary Methods). These images are representative examples of the FACS analysis shown in Fig. 1b.

Supplementary Tables

Supplementary Table S1. List of ωRNA and primers used for amplicon sequencing in this study

Genes	ωRNA	TAM	PCR-F primers (5'- 3')	PCR-R primer (5'-3')
EMX1_Tg1	AATGGTGGAAACACAG	AAGGTA	GCCATATACTTCTGTCTGCTCT	TGTTTGTCAGTTGCGAAATAGC
EMX1_Tg2	GGACATGGCAGATAAT	TAAGTA	ACTACCTCCTCCACATTCTCAG	CACCCTATCCTGGACTTCCTAA
EMX1_Tg3	CCTGACTCTGCAAAGC	CAAGTA	CCTGCCAACGCTGAAGAT	GTTCTAAGCCATCTCCTCCAG
EMX1_Tg4	AATAGCAGATTATTCC	AAAGTA	TCTCCAGGCTCCTGTCCATTCT	TGGCTCCAGCCGATATTTCAGA
EMX1_Tg5	CTAGTCCCTTCCCTTT	AAGGTA	AAATACCTGCCTCCCTGCTAA	AGGAAGAACGCTGGACTAAGA
EMX1_Tg6	AGCTTTTTCCCTGCAG	TTAGAA	GTCGTCATATCTGTCTGCTGC	AAACACTGCCAGGAGGGAAA
VEGFA_Tg1	AAAAGAGTGAACGAGA	CTAGAA	GCCCTCCTCTGTCCCAATT	AGTCCACTGCTTCCTGTGAG
VEGFA_Tg2	ATAGAGCAAGACAAGA	AAAGTA	CATCACCATCTTAACCCTTCCC	GTTGCTGCCACCACAAGTC
VEGFA_Tg3	GGAGGCTCAAAGAGGC	TAAGTA	AGGCTTGGGAACACTTTGAT	TGAGCACTTACTACATCCTGAG

VEGFA_Tg4	CCCTTCAGATCAGCTT	CTAGAA	GAGTAGTCATGCCAGAGTCCT	CTGCCAGCCACTGATAACC
VEGFA_Tg5	GCTGTTCAGGTCTCTG	CTAGAA	GGGTGGATGGCCTAATTC	CTGAACACTGTCTTCCTGCT
VEGFA_Tg6	AAGGCCGCACAGCTAG	TAAGTA	AGGCTTGGGAACACTTTGAT	TGAGCACTTACTACATCCTGAG
ALDH1A3_Tg1	AGTGGAAGAAGGAGAT	AAGGTA	CAACAATGAATGGCACGAATCC	AAGCCGAACCGTTTCCTTATC
ALDH1A3_Tg2	GCTCTGCAGGAACAGG	CAGGTA	CAGACAGGCAGGTGGACAT	GGAGAATGAATGACTGGGAGTG
ALDH1A3_Tg3	TAAAATAAATTTGCTC	TAAGTA	CAACAATGAATGGCACGAATCC	AAGCCGAACCGTTTCCTTATC
ALDH1A3_Tg4	AGAAGGCAGCTTTCTG	CTAGAA	CAGACAGGCAGGTGGACAT	GGAGAATGAATGACTGGGAGTG
ALDH1A3_Tg5	CCAGATTTCTTTTCTC	CTAGAA	CATTTCCCGCTGGTGTGTTT	GTTACCGCTTTCCATGCTCAT
CXCR4_Tg1	CAAAGCCCAAAGTGGT	ATAGAA	TGAACATTCCAGAGCGTGTAGT	TGCTGAAATCAACCCACTCCT
CXCR4_Tg2	AGTGAATCACGTAAAG	CTAGAA	TCTCGTGGTAGGACTGTAGAA	CACTGTAGGTGCTGAAATCAAC
CXCR4_Tg3	TTACAAAATTCTTTGT	TTAGAA	CAGAGGAGTTAGCCAAGATGTG	CAGGCTTGCTTTCTTCAGGAA
PCSK9_Tg1	ATCTTCACCAGGAAGC	CAGGAA	GTGCTGAAGGAGGAGACCC	CCCAGCCCTATCAGGAAGTG

PCSK9_Tg2	AGTGGCCCAGCCCTAT	CAGGAA	TTCCTGGTGAAGATGAGTGG	GGCCAGGCTTAAAGGGA
PCSK9_Tg3	GATGAACCCTCCTTCC	AAGGTA	CACTGCCTGCCTTCCTGTT	GGGCACTGGGAGTTATAGAGG
PRLR	ATTGAAACTGGCAGGG	AAGGAA	AATCTTCCACATGGCCTTTC	GCAGCCTTCTGACTCCCT
Tyr	ATTTGAGTGTCTCCGA	AAAGAA	TTCATGGGTTTCAACTGCG	CATAGGTGCCTGTGGGGAT
Mstn	GACGGTACAAGGTATA	CTGGAA	---	---

Supplementary Table S2. List of primers used for Sanger sequencing in this study

Genes	Species	PCR-F primers (5'- 3')	PCR-R primer (5'-3')
Tyr 5'-arm	Mouse	TATCCTTCTGTCCAGTGCACCATC	AAAGTCCCTATTGGCGTTACTATGG
Tyr 3'-arm		TGGTCCTGCTGGAGTTCGT	CTCCATATTTCAGAGCCCCCA
Mstn	Mouse	ACAGTACAACAAAGTAGTAAAAGCC	ACACTGTCTTCACATCAATACTCTG
PRLR 5'-arm	Cattle	GAAAGTCTGAGAAACAGGAAAGC	GTCCCTATTGGCGTTACTATGG
PRLR 3'-arm		GAGCAAAGACCCCAACGAG	CACATTTGTTGTAAGCTGATACCAT
PRLR GCT>GT	Cattle	TCCCGAAACAAACCTTACATG	AACAGAGCCAGCACTCCATC

Supplementary Table S3. List of primers used for off-target analysis in this study

On-target site	Off-target sites	PCR-F primers (5'- 3')	PCR-R primer (5'-3')
ALDH1A3-Tg1	AGTGGAAGAACGAGAT	GGATGTAGGATGTATGCAT	CTCACAAAGGAGAGTGTC
ALDH1A3-Tg1	AGTGGAAGAAGGAGCT	CCCTGTTCTCTCTTATTTCC	CTTGATAGCTGCTGTGAT
ALDH1A3-Tg1	AGTGGGAGAAGGAGAT	CTGCACATGAACATACACT	GGGTTAAGTTGGTCATAT
ALDH1A3-Tg1	AGTGGAAGATGGAGAT	CATGAAAGCTCAAGCTAC	GATGCTAATTATAAATTGGG
ALDH1A3-Tg1	AGTGGAAGAAAGAGAT	CTCCAGACTTGGGCTTAAG	GGTGCCAGAAAATCATAAG
ALDH1A3-Tg1	AGTGGAAGAAGGAGAG	GCAGTCCCAGCTTACTCAC	ACCCCACATCTCTTTCGT
ALDH1A3-Tg1	AGTGGAAGAAGGAGAG	GAGGGTGTAGGATGACCT	TGTAAGCACCATTACCTG
ALDH1A3-Tg1	AGTGGAAGAAGGATAT	TGGCTAACCCACTGCAAAG	GCTGATCACTGACAATCT
ALDH1A3-Tg1	AGTGGAAGAAGGAGAA	GACCAAGAACATATTCTGC	CCTGTTCTCTTTTCTTCATGC
ALDH1A3-Tg1	AGTGGAAGAAGCAGAT	CTTCAGTGACCCTGGCCTTT	TGTGTTTGAGCTCTGCAG
ALDH1A3-Tg1	AGAGGAAGAAGGAGAT	AAAATGGTATCCTTCACT	CAGTCATACAATCAAATGC

ALDH1A3-Tg1	AGTGGAACAAGGAGAT	TTGTCAACTGTGCATCAG	GGCACTAAATACAAATGC
ALDH1A3-Tg1	AGTGGAAGAAGGAGAC	TAATCTCCACAGCTCTGT	AGGCTTATAACCTTTACTCC
ALDH1A3-Tg1	AGTGGAAGACGGAGAA	AGACAGCTCAGCAGGGTG	CCTACTCTTTAGAAAGGACC
ALDH1A3-Tg1	AGGGGAAGAAGGAGGT	CACATCCAGTTCCATTAT	GTTCATAAAAGGACTGAG
ALDH1A3-Tg1	AGTGGCACAAGGAGAT	TTAGTGGATGCCTGCATT	CCCATTTATCTTTTGTCAAGTCC
ALDH1A3-Tg1	AGTGGAAGGAGGAGAA	TCCTCCAGGTGCTCAGATGT	TGTGTAGGACTGATCCCT
ALDH1A3-Tg1	AGTGGGAGTAGGAGAT	GCTCTCAAACTATATGCTC	GGAATTTACAGGAATGGAG
ALDH1A3-Tg1	AGTGGGAGAAGGAGAA	TTGGTGTATTGGAAACTC	TGGTCCCTCACTCCAGAT
ALDH1A3-Tg1	AGAGGAAGAAGGAGAA	ATCAGTTTCAGGCTTTGCG	GGAGAGAATCGAGAATGGTGTA
EMX1-Tg1	AATGGTGGAAAAACAG	TTCAAACTCCCCTCCTGC	AAGGGAAAAGAAGAGAGG
EMX1-Tg1	AATGGTGGAAGCACAG	CTACTTTAGAGATTGTGCTG	CTATGTGCCAGGTTCTGT
EMX1-Tg1	AATGGTGGGAACACAG	CCAGACTTTATTTAGAGAG	GTGGAGCTGGGGGCATTAT
EMX1-Tg1	GATGGTGGAAACACAG	CTTTCACTGAAGCTGTGT	CCATGAACAAGAGAATGG

EMX1-Tg1	AATGGTGAAAACACAG	GGTCACTTTATTAAATGAC	CCTTTAAGGCTGTCTTGT
EMX1-Tg1	AATGGTGGAAACACTG	TGTTGGCTTCCTGTTCCT	CAGTAGGTGCTGGTGTCT
EMX1-Tg1	AATGCTGGAAACACAG	CACTGTAATTACTGCTAC	TTGCCAAATCTTTAGACGG
EMX1-Tg1	AATGGTGAAAACACAG	GGTCACTTTATTAAATGAC	CCTTTAAGGCTGTTTTGT
EMX1-Tg1	AATGGTGGAAATAAAG	CCTTTAGTAATTACTAACCG	AGCGAATCAAAGCTCTTG
EMX1-Tg1	AGTGGTGGTAACACAG	TTACATCGTGGCGTGTTC	CCTGTGGTTTTCTCTGGT
EMX1-Tg1	AAAGGTGGACACACAG	TCGGGACTGTAGGCTGGTT	TCAACTGCTCCATCTCAT
EMX1-Tg1	AATGATGGAAAGACAG	ATAGTTAGGCACTCAGGC	CCTGAAGAAGAATGAATC
EMX1-Tg1	AATGGAGGAAACACAT	GGCTTGACAACAGTCTTT	CTCACATGGAGTTGGTAC
EMX1-Tg1	AATAGTGGAAACAGAG	TCCCAAGCGTCTATTCCT	GGGTCTATCTAAATGGCC
EMX1-Tg1	AATGGGGGAAACCCAG	CCTACCTGTCCTTTCAAT	AATCCCAGCTGCTATTCT
EMX1-Tg1	AATGGTGGAAAGACTG	GGAGACTAAGGAGATCTAAC	GGTACAGTTATCAAAGCC
EMX1-Tg1	AATTGTGGAAAAACAG	CTGTATTTACCTGTTCGTAG	CTCATACAGAACTCAATC

EMX1-Tg1	CATGGTGGAAACTCAG	CAGCTCATGACCAAAGAC	CTATGAATTTTGTAGTCC
EMX1-Tg1	AATGGTGGGAACCCAG	CACTGTGGTCCCCAGAGA	TAGTGACCCCCTTTGTGG
EMX1-Tg1	AATGGTGGAAGCAGAG	ATCAGTGTACTGGCGGAG	TGCTTACCTGGAAAGCCC
VEGFA-Tg2	ATAGAGCAACACAAGA	CCTGGAAACCTCAACTTT	CACTTGAACAATGGTGATG
VEGFA-Tg2	GTAGAGCAAGACAAGA	ACAGGGAGGGGTGAATAT	GAGCAAACCTATCTAGAG
VEGFA-Tg2	ATAGAGCAAGACAAAA	GTATTCATTTCCTTATTC	GATCACTTGAGCGCTGTAGT
VEGFA-Tg2	ATAGAGCAAGACAACA	CCCATGGGAGATGATTAT	TTCAGCTGGGAAACTTAG
VEGFA-Tg2	ACAGAGCAAGACAAGA	TCGCTCTTTCCCTCTCTCT	CAGGGGAATCACTTGAAG
VEGFA-Tg2	ATAGAGCAAGACAAAA	GTTTTCTTAGTTTCTGGG	CCATGAATGCACAATGCAAT
VEGFA-Tg2	ACAGAGCAAGACAAGA	GTGGCCCTAGTTGCGTTT	TTGAATCTGGGAGGTGGAG
VEGFA-Tg2	ATAGAGCAAGACAACA	GGAAGTGACTCCTGGAGA	AGATAACCGATGTGAAGG
VEGFA-Tg2	ATAGAGCAAGACAACA	TCATGGACACTAGAGCTT	CTCGAGTTTCTTTTTAACC
VEGFA-Tg2	ACAGAGCAAGAGAAGA	CTGTTTCCCCCCTTTCTT	TTGTGCAGCTTTAGGCAT

VEGFA-Tg2	ATAGAGCAAAAGAAGA	GGAAATTTCTTCCCAGAT	TCAACTGCCGTGTGATAT
VEGFA-Tg2	ATAGAGCAAGACCCGA	CTTTCTTTCTTTCCTTCTCTCTC	ATCACGTAAGCCCAGGAGT
VEGFA-Tg2	CTAGAGAAAGACAAGA	GGTTACCGCAGCAAACAC	GCTTTCACAGCATTTACT
VEGFA-Tg2	ATAAAGCAAGAAAAGA	TTCCTTCTTTTCCTGCAG	GGAAGATGCAGAAATAAA
VEGFA-Tg2	ATAGAGCAAGACTGGA	AGCCCAGGAGTTTGAGGT	CAATGGCTTCAAGATTCTC
VEGFA-Tg2	ATAGAACAAGACAAGC	TATTATACTGACCTCACC	CCCAACAGTCACAATTTAT
VEGFA-Tg2	AGAGAGCAAGCCAAGA	CAAGGACATTCCCTTTGT	GCAAGGGATCAAACAATT
VEGFA-Tg2	ATAGAGCAAGAAATGA	TAATGTGTGAAGCAGCAG	GATTTTCATCTGTCTTGG
VEGFA-Tg2	ATAGAGAAAGAAAAGA	GGGAGAGTACATTTTCATG	GGCAGACACTGTGAGGATT

Supplementary Methods

Animals and chemicals

All animal procedures were performed under the ethical guidelines of the State Key Laboratory of Reproductive Regulation and Breeding of Grassland Livestock, College of Life Sciences, Inner Mongolia University (IMU). Specific pathogen-free-grade C57BL/6 and CD1 mice were purchased from Laboratory Animal Research Center (Inner Mongolia University) or Vital River Laboratories Co., Ltd. Cattle are raised at the experimental farm of IMU and processed in commercial slaughterhouses. Unless otherwise indicated, all chemicals were purchased from Sigma-Aldrich Chemical Reagent Co., Ltd.

Plasmid construction

The IscB$^{\#}$, ωRNA$^{\#}$ scaffold, EGXXFP, EGFP, and SLICK-PRLR sequences were designed and synthesised by Sangon Biotech Co., Ltd., based on previous reports [1, 3, 5]. These DNA sequences were inserted into the pEJS1089 AAV backbone (Addgene, 159536). Overall, the AAV::IscB$^{\#}$/ωRNA$^{\#}$ plasmid encoded the optimised IscB with one SV40 nuclear localisation signal (NLS) on the N terminus and one nucleo-plasmin NLS on the C terminus driven by U1a promoter, and U6-driven ωRNAs with or without HDR-template. The AAV::IscB$^{\#}$/ωRNA$^{\#}$-HDR sequences are shown in Supplementary Fig. S16.

Cell culture and plasmid transfection

HEK293T and HeLa cells were cultured in Dulbecco's Modified Eagle Medium (DMEM, Gibco) supplemented with 10% fetal bovine serum (FBS, Gibco) and 1% Penicillin/Streptomycin (Gibco) at 37 °C with 5% CO_2. Cells were seeded in a 12-well plate at approximately 2×10^5 cells/well and transfected after 24 hours at ~70% confluency. A total of 2 μg plasmids were transfected by Lipofectamine-3000 (Invitrogen) according to the manufacturer's protocol. Cells were collected 72 hours

after transfection for further analysis. The ωRNA sequences are listed in Supplementary Table S1.

FACS analysis

Fluorescence-activated cell sorting (FACS) was performed as previously described [3]. Briefly, cells were washed with phosphate-buffered saline (PBS) and digested with 0.25% Trypsin-EDTA (Gibco) at 37 °C. After 5 minutes, the cells were resuspended with cold PBS at approximately 2×10^6 cells/mL, and passed through 35 μm cell strainer. The resuspended cells were analyzed or sorted by FACSAria II (BD biosciences). Results were analyzed using FlowJo (Ashland). The example gating scheme is shown in Supplementary Fig. S17.

Zygotes preparation

Mouse and bovine zygotes were produced according to previous reports [6, 7]. For mouse zygote collection, mice were housed in a 12-hour dark/12-hour light cycle; zygotes were collected at embryonic day 0.5 (E0.5) by tearing the ampulla with the ophthalmic tweezers in the M2 medium. For bovine zygote preparation, we use ovaries from Angus or Simmental cattle obtained from the slaughterhouse and cryopreserved sperm from the same breeds. The IVF procedure was conducted in the BO-IVF medium (IVF Bioscience) with approximately 2×10^6 sperm/mL for 15 hours at 38.5 °C with 5% CO2.

AAV production and infection

Packaging of AAV vectors was performed by GenePharma Co., Ltd., based on previous reports [8, 9]. Zygote infection was conducted according to the published protocols with slight modifications [7, 8]. Briefly, mouse or bovine intact zygotes (without removing the zona pellucida) were placed in 20 μL drops of KSOM-AA (Millipore) containing 5×10^8 to 5×10^{10} VG/mL AAV for 12 to 36 hours (based on the

experimental design). After the incubation period, the embryos were rinsed once in M2 medium and transferred to the G1/G2 medium (1:1; Vitrolife) for subsequent culture.

Pronuclear microinjection

Generation of gene-edited embryos via pronuclear microinjection was conducted as per previously published studies [8, 10]. In vitro transcription of IscB# and ωRNA# was performed using the mMESSAGE mMACHINE (Invitrogen) and MEGAshortscript Kit (Invitrogen), respectively, according to the manufacturers' recommendations. For microinjection, superovulated female C57BL/6 mice were mated with males of the same strain, and fertilized zygotes were harvested from the oviducts at E0.5. Based on previously reported injection concentrations [10], a mixture of IscB# mRNA (100 ng/μL) and one or two ωRNA# (100 ng/μL each) was prepared. The distinct male pronucleus of zygotes was microinjected in M2 medium droplets using a FemtoJet microinjector (Eppendorf) with a constant flow setting. Post-injection, zygotes were cultured in KSOM-AA (Millipore) for 3 days until reaching the blastocyst stage, after which they were subjected to genotype analysis.

Embryo transfer

For mouse embryo transfer, the 2-cell stage embryos were transferred to the oviducts of E0.5 pseudo-pregnant (~ 20 embryos per mouse) according to our previous report [11]. For bovine embryo transfer, the blastocysts were transferred via the non-surgical transcervical method as previously described [12]. Briefly, a single blastocyst was transferred into the uterine horn of hormonally synchronised (CIDR progesterone + cloprostenol + gonadorelin) nulliparous heifer at day 7, estrus day 0 = day of AAV infection. Pregnancy detection was diagnosed on day 35 using the transrectal ultrasound EVO scanner (5.0 MHz linear probe, E.I. Medical Imaging).

Amplicon sequencing and data analysis

Next-generation sequencing (NGS) and data analysis were followed by previously published studies [3, 8, 13]. Briefly, the genomic DNA was extracted from transfected cells, infected embryos, and cattle ear-tips using the TIANamp Genomic DNA Kit (Tiangen) or QIAamp DNA Mini Kit (Qiagen) according to the manufacturer's instructions. Genomic sites of interest were amplified using unique barcoding primer pairs with Phanta Max Super-Fidelity DNA Polymerase (Vazyme) for deep sequencing. The PCR products were pooled by common amplicons and sequenced with pair-end 150 bp on the HiSeq 4000 system (Illumina) by Annoroad Gene Technology Co., Ltd. Data analysis was performed using CRISPResso2 software [14]. The editing efficiencies were quantified as the percentage of total sequencing reads containing indels. Primers used for NGS are listed in Supplementary Table S1.

TOPO sequencing and data analysis

The PCR amplification was performed using Phanta Max Super-Fidelity DNA Polymerase (Vazyme) with specific primers. The amplicon was cloned into the pCE3 plasmid using the Ultra-Universal TOPO Cloning Kit (Vazyme) and transformed *E. coli* DH5α competent cells, according to the manufacturer's instruction. The plasmids from individual 50 colonies underwent Sanger sequencing at Sangon Biotech Co., Ltd., and the results were analyzed using TIDE software. Primers used for Sanger sequencing are listed in Supplementary Table S2.

TOPO sequencing and data analysis

The PCR amplification of target loci was performed using Phanta Max Super-Fidelity DNA Polymerase (Vazyme) with locus-specific primers. The PCR product was purified using the QIAquick PCR Purification Kit (Qiagen) to remove residual primers and dNTPs. The purified amplicon was cloned into the pCE3 plasmid using the Ultra-Universal TOPO Cloning Kit (Vazyme) following the manufacturer's protocol, and transformed into *E. coli* DH5α competent cells. After overnight culture on LB agar

plates containing appropriate antibiotics, 50 individual colonies were randomly picked for plasmid extraction using the FastPure Plasmid Mini Kit (Vazyme). The extracted plasmids were subjected to Sanger sequencing at Sangon Biotech Co., Ltd. For data analysis, sequencing results were aligned to the wild-type reference sequence of the target locus. Clones harboring the expected HDR events (e.g., specific point mutations or gene insertions) were identified as HDR-positive clones. The percentage of TOPO-clones was calculated as (number of HDR-positive clones / total Sanger-sequenced clones) × 100. Raw sequencing data were analyzed using TIDE software to verify mutation accuracy and exclude false-positive signals [15]. Primers used for Sanger sequencing are listed in Supplementary Table S2.

Off-target sites prediction

Off-target sites were predicted using the Cas-OFFinder software, all the parameters were set as in the previous report [3]. Briefly, the search queries were set to cover the 16-nt on-target ωRNA spacer and 'NWRRNA'. The mismatches and PAM were set to 3 and 'NNN', respectively. Indeed, the predicted off-target sites of the top 20 were selected to design primers. Primers used for off-target analysis are listed in Supplementary Table S3.

Statistical analysis

GraphPad Prism software was used to analyze the data. The numerical values are presented as mean ± S.E.M. or S.D., as indicated in the legend. Differences between control and treatments were tested using two-tailed Student's *t*-tests, n ≥ 3 independent experiments.

Data availability

The sequencing data that support the findings of this study have been deposited in the

Gene Expression Omnibus (GEO) under accession codes GSE308343 and GSE308344. All materials are available upon reasonable request.

Supplementary References

1 Mashiko D, Fujihara Y, Satouh Y, Miyata H, Isotani A, Ikawa M. Generation of mutant mice by pronuclear injection of circular plasmid expressing Cas9 and single guided RNA. *Sci Rep* 2013; **3**:3355.

2 Altae-Tran H, Kannan S, Demircioglu FE *et al.* The widespread IS200/IS605 transposon family encodes diverse programmable RNA-guided endonucleases. *Science* 2021; **374**:57-65.

3 Han D, Xiao Q, Wang Y *et al.* Development of miniature base editors using engineered IscB nickase. *Nat Methods* 2023; **20**:1029-1036.

4 Certo MT, Ryu BY, Annis JE *et al.* Tracking genome engineering outcome at individual DNA breakpoints. *Nat Methods* 2011; **8**:671-676.

5 Littlejohn MD, Henty KM, Tiplady K *et al.* Functionally reciprocal mutations of the prolactin signalling pathway define hairy and slick cattle. *Nat Commun* 2014; **5**:5861.

6 Yang L, Song L, Liu X, Bai L, Li G. KDM6A and KDM6B play contrasting roles in nuclear transfer embryos revealed by MERVL reporter system. *EMBO Rep* 2018; **19**:e46240.

7 Krivonogova AS, Bruter AV, Makutina VA *et al.* AAV infection of bovine embryos: Novel, simple and effective tool for genome editing. *Theriogenology* 2022; **193**:77-86.

8 Yoon Y, Wang D, Tai PWL, Riley J, Gao G, Rivera-Perez JA. Streamlined ex vivo and in vivo genome editing in mouse embryos using recombinant adeno-associated viruses. *Nat Commun* 2018; **9**:412.

9 Mizuno N, Mizutani E, Sato H *et al.* Intra-embryo gene cassette knockin by CRISPR/Cas9-mediated genome editing with adeno-associated viral vector. *iScience* 2018; **9**:286-297.

10 Wang M, Sun Z, Liu Y *et al.* Hypercompact TnpB and truncated TnpB systems enable efficient genome editing in vitro and in vivo. *Cell Discov* 2024; **10**:31.

11 Yang L, Liu X, Song L *et al.* Transient Dux expression facilitates nuclear transfer and induced pluripotent stem cell reprogramming. *EMBO Rep* 2020; **21**:e50054.

12 Owen JR, Hennig SL, McNabb BR *et al.* One-step generation of a targeted knock-in calf using the CRISPR-Cas9 system in bovine zygotes. *BMC Genomics* 2021; **22**:118.

13 Anzalone AV, Randolph PB, Davis JR *et al.* Search-and-replace genome editing without double-strand breaks or donor DNA. *Nature* 2019; **576**:149-157.

14 Clement K, Rees H, Canver MC *et al.* CRISPResso2 provides accurate and rapid genome editing sequence analysis. *Nat Biotechnol* 2019; **37**:224-226.
15 Brinkman EK, Chen T, Amendola M, van Steensel B. Easy quantitative assessment of genome editing by sequence trace decomposition. *Nucleic Acids Res* 2014; **42**:e168.
